# Treatment decisions, clinical outcomes, and pharmacoeconomics in the treatment of patients with *EGFR* mutated stage III/IV NSCLC in Germany: an observational study

**DOI:** 10.1186/s12885-018-4032-3

**Published:** 2018-02-05

**Authors:** Wolfgang Schuette, Peter Schirmacher, Wilfried E. E. Eberhardt, Manfred Dietel, Ute Zirrgiebel, Lars Muehlenhoff, Michael Thomas

**Affiliations:** 1 Krankenhaus Martha-Maria Halle-Doelau gGmbH, Klinik für Innere Medizin II, Roentgenstr, 106120 Halle, Germany; 2 Pathologisches Institut, Universitätklinik Heidelberg, Heidelberg, Germany; 3 Department of Medical Oncology, West German Tumor Centre, University Hospital Essen, Rurhlandlkinik, University Duisburg-Essen, Essen, Germany; 4 Pathologisches Institut Humboldt, Universität Berlin, Berlin, Germany; 5 iOMEDICO AG, Freiburg, Germany; 6 Medical Affairs, AstraZeneca, Wedel, Germany; 7 Internistische Onkologie der Thoraxtumoren, Thoraxklinik im Universitätsklinikum Heidelberg, Translational Lung Research Center Heidelberg (TLRC-H), Member of the German Center for Lung Research, Heidelberg, Germany

**Keywords:** EGFR-mutations, Non-small cell lung cancer (NSCLC), EGFR tyrosine kinase inhibitor, Observational, REASON study

## Abstract

**Background:**

We evaluated treatment decisions and outcomes in a cohort of predominately Caucasian patients with *EGFR* mutation-positive (*EGFR* Mut+) non-small-cell lung cancer (NSCLC).

**Methods:**

REASON (NCT00997230) was a non-interventional study in German patients with stage IIIB/IV NSCLC. Secondary endpoints for *EGFR* Mut + NSCLC included progression-free survival (PFS), overall survival (OS), adverse event (AE) management, and pharmacoeconomic outcomes.

**Results:**

Among 334 patients with *EGFR* Mut + NSCLC, tyrosine kinase inhibitors (TKIs) were the most common first-line therapy (56.6%, 53.0% gefitinib). Among patients who received TKIs/gefitinib before first disease progression, PFS was longer compared with those who did not receive a TKI (median 10.1/10.0 vs. 7.0 months; HR 0.67/0.69; log-rank *p* = 0.012/*p* = 0.022). OS was longer for those patients who ever received a TKI/gefitinib during their complete therapy course compared with those who never received a TKI (median 18.4/18.1 vs. 13.6 months; HR 0.53/0.55; *p* = 0.003/*p* = 0.005). Total mean first-line treatment healthcare costs per person were higher for those receiving TKIs (€46,443) compared with those who received chemotherapy (€27,182). Mean outpatient and inpatient costs were highest with chemotherapy. Rash, diarrhea, and dry skin were the most commonly reported AEs for patients receiving gefitinib.

**Conclusions:**

In REASON, TKI therapy was the most common first- and second-line treatment for *EGFR* Mut + NSCLC, associated with increased drug costs compared with chemotherapy. Patients who received gefitinib or a TKI ever during their complete therapy course had prolonged PFS and OS compared with patients who did not receive a TKI.

**Trial registration:**

The trial was registered on October, 2009 with ClinicalTrials.gov: https://clinicaltrials.gov/ct2/show/NCT00997230?term=NCT00997230&rank=1

**Electronic supplementary material:**

The online version of this article (10.1186/s12885-018-4032-3) contains supplementary material, which is available to authorized users.

## Background

Non-small cell lung cancer (NSCLC) accounts for 85–90% of lung cancers [1]. Among those patients with NSCLC, mutations in the epidermal growth factor receptor (EGFR) are present in 30–40% of Asian patients and 10–20% of white patients [2]. EGFR tyrosine kinase inhibitors (TKIs), such as gefitinib have demonstrated efficacy compared with chemotherapy in patients with locally advanced or metastatic NSCLC with activating mutations of the TK domain of the EGFR [3, 4]. *EGFR* testing is now a standard approach in the work-up of patients with advanced NSCLC and is recommended by the ESMO Clinical Practice European guidelines and German lung cancer guidelines [1, 5].

The primary aim of this non-interventional study, Registry for the Epidemiological and Scientific evaluation of *EGFR* mutation status in patients with newly diagnosed locally advanced or metastatic NSCLC (REASON), was to generate data on *EGFR* mutation status from a large cohort of predominantly Caucasian patients and to correlate it with clinicopathological characteristics. Detailed primary endpoint results from REASON are reported in a separate publication [6]. In summary, among 4200 evaluable patients, 431 (10.3%) had *EGFR* mutation-positive (Mut+) disease. The odds of *EGFR* mutation were significantly higher (*P* < 0.0001) in females versus males (odds ratio 1.85; 95% confidence interval 1.48, 2.32), never smokers versus ever smokers (3.64; 2.91, 4.56), and adenocarcinoma versus other histological sub-types (2.94; 2.17, 4.08).

In this paper, we report the results for the secondary endpoints of REASON, including detailed analyses of treatment decisions, clinical outcome, safety and tolerability (restricted to patients with *EGFR* Mut + NSCLC who received gefitinib), and pharmacoeconomic outcomes. We also report explorative analyses of clinical outcomes in patients with *EGFR* Mut + NSCLC who received gefitinib, which was the most commonly prescribed first-line EGFR-TKI.

## Methods

The study design has been reported in detail elsewhere [6]. Briefly, this was a national, multicenter, prospective, observational study carried out in 149 centers in Germany in patients with newly diagnosed stage IIIB/IV NSCLC (NCT00997230). Patients were treated and assessed under real-life conditions and data were taken from the electronic case report form.

Given the non-interventional design of the study, intervals for follow-up were conducted according to the routine practice of the centers. Responses were documented according to the radiologist’s report (and not according to pre-specified criteria) and could be radiological or clinical, as judged by the investigator. Formal Response Evaluation Criteria In Solid Tumors (RECIST) was not performed.

Patients were ≥18 years with histologically confirmed stage IIIB/IV NSCLC and suitable for first-line treatment, but not amenable to curative surgery or radiotherapy, and with suitable tumor tissue available for *EGFR* testing [6]. Participation was until documentation of the first-line treatment decision. Patients with *EGFR* Mut + NSCLC receiving first-line therapy, and not participating in other interventional studies, could continue until patients’ decision to withdraw, death, or loss to follow-up.

### Endpoints

The primary endpoint of the study has been reported previously [6]. Secondary endpoints were analyzed only for patients with *EGFR* Mut + disease who were not participating in other clinical trials, with the exception of first-line treatment decisions and concomitant therapy, which were investigated in all patients.

Treatment decisions were recorded for first-line and planned second-line treatments. Multiple agents could be recorded for treatment decisions. Amendments to the protocol allowed for extended data capture (subject to consent of patients and data cut-off at 31 October 2012): documentation of actual treatments beyond first-line, extension of follow-up until patient’s death, and retrospective documentation of the date of death for all patients with *EGFR* Mut + disease (as assessed by Ethics Committee).

Clinical outcome records included progression-free survival (PFS), overall survival (OS), and response rate (RR) (complete response plus partial response). Disease control rate was originally designated as an endpoint but could not be determined due to the unknown duration of stable disease resulting from the lack of a standardized frequency of follow-up documentation.

Reported adverse events (AEs) for supportive treatments and AE management associated with first-line treatment in patients receiving gefitinib were recorded. AEs reported more than once for a patient, and with at least one occurrence considered by the physician to be gefitinib related, were classified as adverse drug reactions (ADRs). AEs were graded according to the National Cancer Institute Common Terminology Criteria for Adverse Events Version 3.0.

Resource use and costs were analyzed for first-line drug therapy (based on type and duration of therapy and priced using the LAUER-TAXE® price list, a German price list reflecting the official prices for prescribed pharmaceuticals). Outpatient care costs were based on the number of outpatient visits according to the physicians’ specialty and services used, and calculated using the Doctors’ Fee Scale within the Statutory Health Insurance Scheme (Einheitlicher Bewertungsmaẞstab). Inpatient care costs were based on the number of inpatient stays and the number of days in hospital associated with the event and calculated using the national Diagnosis-Related Groups for inpatient services. Auxiliary nursing support and incapability to work (based on changes between baseline and end of the observation period) were also recorded; however, no costs were assigned to these.

### Statistical methods

Descriptive statistics were used with 95% confidence limits. Binary, categorical, and ordinal parameters were summarized by means of absolute numbers and percentages (including ‘missing data’ as a valid category). Statistical tests, which were performed two-sided at a 5% level of significance, were descriptive-exploratory.

A multivariate logistic regression analysis of factors influencing first-line therapy decisions (TKI vs. no TKI) was conducted including: mutational status known at therapy initiation, age, gender, smoking status, tumor histology, disease status at diagnosis, Eastern Cooperative Oncology Group performance status, tumor stage, and tumor grade (Grade 1 [well differentiated] to Grade X [cannot be assessed]). For clinical outcomes, analysis was performed by receipt of TKI/gefitinib vs. no TKI. The Kaplan-Maier-method was used to estimate PFS and OS. Patients without an event at data cut-off were censored cases.

For pharmacoeconomic analyses, descriptive statistics for the costs were computed for continuous variables over the observation period. Subgroup analysis was performed according to therapy received (chemotherapy or TKI) in the first-line setting, including those patients who switched therapy.

## Results

Of 4243 patients enrolled into the study, baseline documentation was available for 4200 of which 4196 fulfilled all inclusion criteria with a total of 431 (10.3%) patients tested positive for *EGFR* Mut + tumors. The disposition of patients through the study has been previously reported [6]. Documented decision of first-line treatment was collected for 2946 patients (69%; 2481 *EGFR* mutation-negative [Mut-; 58%], 131 *EGFR* Mut unknown [3%], and 334 *EGFR* Mut + [7%]). The majority of patients (84.9%) were treated in a hospital (81.7% and 85.2% of patients with *EGFR* Mut + and *EGFR* Mut- disease, respectively): 59.8% inpatients, 27.8% outpatients, and 12.4% daytime care. A further 14.3% of patients were treated by an oncologist in private practice and 0.8% of patients were treated by a pneumologist. During this study, a greater proportion of patients with *EGFR* Mut- disease were treated as inpatients (63.7%) compared with patients with *EGFR* Mut + disease (32.6%).

The most common first-line treatments selected were carboplatin (45.5%), cisplatin (33.9%), and pemetrexed (28.2%) (Table 1). TKIs/gefitinib were received as first-line therapy in 8.2%/6.2% of all patients and 56.6%/53.0% of patients with *EGFR* Mut + NSCLC (*n* = 334). Combination chemotherapy, generally platinum-based, was received in 35.0% of patients with *EGFR* Mut + disease; 78.5% of *EGFR* Mut- patients received combination chemotherapy and 12.9% received monochemotherapy. The most commonly used agents for patients with *EGFR* Mut- disease were carboplatin (48.5%), cisplatin (36.2%), and pemetrexed (30.4%).

**Table 1:** First-line treatment decisions

*n*, %	*EGFR* Mut+ *n* = 334	*EGFR* Mut- *n* = 2481	*EGFR* Mx *n* = 131	Total *N* = 2946
Agent				
Carboplatin	74 (22.2)	1203 (48.5)	62 (47.3)	1339 (45.5)
Cisplatin	60 (18.0)	897 (36.2)	43 (32.8)	1000 (33.9)
Pemetrexed	39 (11.7)	754 (30.4)	38 (29.0)	831 (28.2)
Gemcitabine	37 (11.1)	603 (24.3)	36 (27.5)	676 (22.9)
Vinorelbine	43 (12.9)	586 (23.6)	41 (31.3)	670 (22.7)
Paclitaxel	21 (6.3)	284 (11.4)	8 (6.1)	313 (10.6)
Gefitinib	177 (53.0)	6 (0.2)	0	183 (6.2)
Bevacizumab	18 (5.4)	142 (5.7)	1 (0.8)	161 (5.5)
Docetaxel	3 (0.9)	97 (3.9)	1 (0.8)	101 (3.4)
Etoposide	1 (0.3)	76 (3.1)	3 (2.3)	80 (2.7)
Erlotinib	12 (3.6)	46 (1.9)	3 (2.3)	61 (2.1)
Other	0	19 (0.8)	1 (0.8)	20 (0.7)
Cetuximab	0	3 (0.1)	0	3 (0.1)
Type of treatment				
Combination chemotherapy	117 (35.0)	1947 (78.5)	103 (78.6)	2167 (73.6)
Monochemotherapy^a^	10 (3.0)	319 (12.9)	23 (17.6)	352 (11.9)
TKI	189 (56.6)	49 (2.0)	3 (2.3)	241 (8.2)
Chemotherapy + bevacizumab and/or cetuximab	18 (5.4)	141 (5.7)	1 (0.8)	160 (5.4)
Not classifiable^b^	0	19 (0.8)	1 (0.8)	20 (0.7)
Other	0	6 (0.2)	0	6 (0.2)

Patients with at least one specification of chemotherapy – multiple answers were permitted. Individual agents and treatment type ranked in order of decreasing use in the total population. Mut+, mutation-positive; Mut-, mutation-negative; Mx, mutation unknown/non-evaluable; TKI, tyrosine kinase inhibitor. ^a^Carboplatin, cisplatin, docetaxel, etoposide, gemcitabine, paclitaxel, pemetrexed, vinorelbine. ^b^Therapy schemes included ‘other’ substances (from free text entries)

At follow-up, 58.8%/55.0% of 320 patients with *EGFR* Mut + NSCLC had received TKI/gefitinib therapy, 21.9% were receiving combination chemotherapy, and 10.0%/9.4% had switched from combination chemotherapy to TKI/gefitinib therapy. First-line therapy was continued as maintenance in 71 (22.2%) patients with *EGFR* Mut + NSCLC, mainly planned to be gefitinib (44 patients). There was an indication that older patients were more likely to receive TKIs than younger patients (odds ratio 1.05, 95% CI 1.01–1.09, *P* = 0.01). Reasons why patients did not receive a TKI were not collected.

**Fig. 1:**
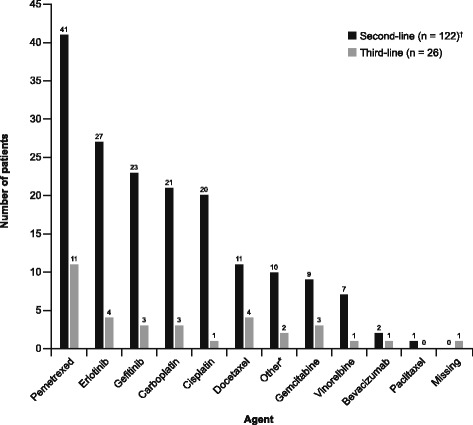
Second- and third-line treatment in patients with *EGFR* Mut + NSCLC More than one agent could be reported. *Other = experimental (*n* = 4 s-line), afatinib (*n* = 3 s-line), experimental afatinib (*n* = 2 third-line), gefitinib/placebo (*n* = 1 s-line), trofosfamide (*n* = 1 s-line). ^†^Data for patients receiving second-line treatment are a combination of planned treatment (*n* = 63 patients who did not consent to collection of data for second-line treatment) and actual treatment (*n* = 59 patients who consented to collection of data for second and subsequent lines of treatment). Mut+, mutation-positive; NSCLC, non-small-cell lung cancer

The most common second-line therapy choice among 122 patients with *EGFR* Mut + disease was TKI therapy followed by pemetrexed and platinum agents (Fig. 1). Nine patients received second-line treatment within a clinical study. Among the 26 patients receiving third- and subsequent-line treatment, pemetrexed was the most commonly used treatment, followed by a TKI (Fig. 1). Of the 320 *EGFR* Mut + patients with follow-up visits, 242/213 had documented TKI/gefitinib treatment (17 documented as planned TKI treatment). No TKI treatment was documented for 61 patients during the REASON study.

### Clinical outcomes

Of the 334 patients with *EGFR* Mut + disease and documented first-line treatment, 320 were assessed for clinical outcome, of which 220/206 had received a TKI/gefitinib during first-line treatment. The mean number of documented tumor evaluations per patient was 4.9 among those receiving first-line TKIs and 4.1 among those not receiving TKIs.

Among the 320 patients assessed for clinical outcome, the estimated median OS and PFS was 17.2 months and 9.1 months, respectively (Table 2). Among groups of patients analyzed, OS and PFS were longer in the following: female versus male; never smoker versus ever smoker (Table 2). Additionally, PFS was longer in the following: adenocarcinoma versus non-adenocarcinoma; TKI-sensitive versus TKI-insensitive *EGFR* mutations.

**Table 2:** OS, PFS, and RR in patients with *EGFR* Mut + NSCLC

	n	Overall survival		Progression-free survival		Response rate	
		Median (months)	95% CI	Median (months)	95% CI	n	%
Overall	320	17.2	15.1–19.8	9.1	8.5–10.3	163	50.9
Gender							
Female	200	20.4	17.2–23.8	10.3	9.4–12.6	110	55.0
Male	120	12.2	9.6–17.0	6.8	5.1–8.8	53	44.2
		*P* < 0.001^a^		*P* < 0.001^a^			*P* = 0.078^b^
Histology							
Adenocarcinoma	286	17.0	15.1–19.5	9.3	8.7–10.5	148	51.7
Non-adenocarcinoma	33	18.4	12.2–NA	6.9	5.1–21.3	15	45.5
				*P* = 0.82^a^			*P* = 0.616^b^
Smoking habit							
Ever smoker	168	15.1	13.6–18.1	8.1	6.8–10.3	79	47.0
Never smoker	150	20.4	17.0–26.5	10.2	9.1–12.0	83	55.3
		*P* = 0.014^a^		*P* = 0.029^a^			*P* = 0.172^b^
First-line therapy							
Ever EGFR inhibitor	220	16.4	14.3–20.3	9.6	8.8–11.1	118	53.6
No EGFR inhibitor	100	18.1	15.1–23.5	8.7	6.3–11.2	45	45.0
Ever gefitinib	206	16.4	14.2–20.4	9.6	8.6–10.9	111	53.9
TKI	188	17.4	14.7–20.4	9.7	8.5–11.4	100	53.2
Gefitinib	176	17.4	14.7–20.4	9.6	8.1–11.3	94	53.4
Chemotherapy	100	18.1	15.1–23.5	8.7^c^	6.3–11.2	45	45.0
Chemotherapy → TKI	32	13.9	9.1–NA	9.2	8.6–21.6	18	56.3
Chemotherapy → gefitinib	30	10.3	8.6–21.6	13.8	8.6–NA	17	56.7
TKI maintenance planned	57	19.8	15.0–NA	10.3	8.7–16.3	38	66.7
No TKI maintenance planned	263	16.4	14.2–19.1	9.0	7.7–10.3	125	47.5
TKI from start	158	16.4	13.1–20.3	9.7	7.6–11.4		
Change to TKI/planned TKI maintenance	76	17.9	14.8–NA	10.0	8.7–14.8		
No TKI	86	18.0	14.2–22.5	8.1	6.1–11.2		
TKI treatment^d, e^							
TKI from start	188	17.4	14.7–20.4	9.7	8.5–11.4		
TKI switch/planned maintenance	46	17.0	10.0–NA	10.0	8.6–21.4		
No TKI (first + maintenance)	86	18.0	14.2–22.5	8.1	6.1–11.2		
TKI documented	229	17.9	15.0–20.5	10.1	8.9–11.7		
Gefitinib documented	206	17.4	14.8–20.4	10.0	8.8–11.4		
Planned TKI documented	12	NA	NA	8.7	3.6–NA		
No TKI documented	79	15.4	13.8–22.5	7.0	5.1–9.4		
TKI treatment^e, f^							
TKI documented	242	18.4	16.3–21.8				
Gefitinib documented	213	18.1	15.5–21.4				
Planned TKI documented	17	17.0	10.0–NA				
No TKI documented	61	13.6	9.3–15.4				
*EGFR* mutation							
TKI-sensitive	231	18.1	15.5–20.9	10.2	9.1–11.7	132	57.1
TKI-insensitive	24	17.9	6.9–NA	5.4	4.0–9.4	8	33.3
							*P* = 0.044^b^

OS, PFS, and RR by demographic and clinico-pathological characteristics, and therapy in patients with EGFR Mut + NSCLC. ^a^Log-rank test. ^b^Chi-squared test. ^c^Includes two patients in whom the therapeutic agent was changed within first-line treatment but the new agent was not documented. ^d^TKI until first documented tumor progression. ^e^Analysis not prespecified. ^f^Patients who ever received a TKI as part of their complete therapy course

CI, confidence interval; NA, not available; NSCLC, non-small-cell lung cancer; RR, response rate; OS, overall survival; PFS, progression-free survival; TKI, tyrosine kinase inhibitor

**Fig. 2:**
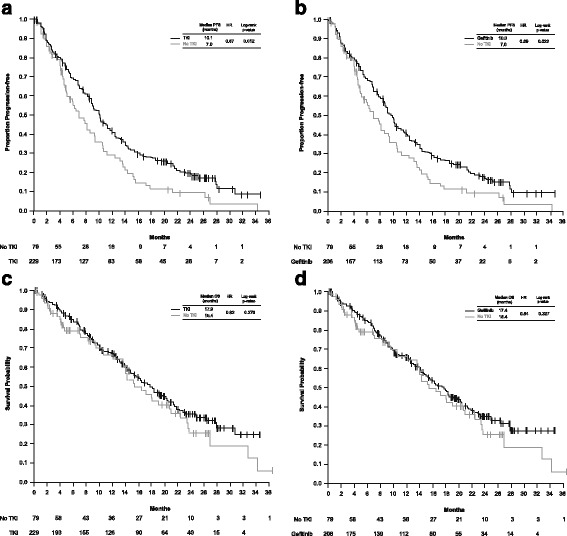
KM estimates of PFS and OS: patients with *EGFR* Mut + advanced NSCLC by therapy Kaplan-Meier estimates of progression-free survival (**a** and **b**) and overall survival (**c** and **d**), of patients with *EGFR* Mut + advanced NSCLC who received either a TKI (**a** and **c**) or gefitinib (**b** and **d**) prior to first disease progression compared with those patients who did not receive a TKI prior to first disease progression. KM, Kaplan-Meier; Mut+, mutation-positive; NSCLC, non-small-cell lung cancer; TKI, tyrosine kinase inhibitor

**Fig. 3:**
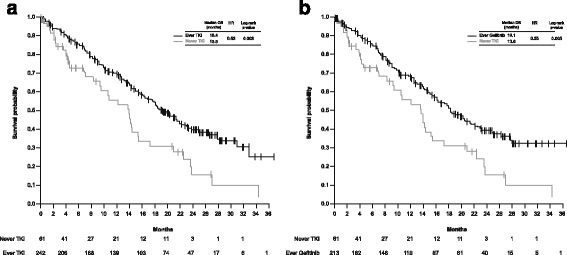
KM estimates of OS: patients with *EGFR* Mut + advanced NSCLC who ever received a TKI Kaplan-Meier estimates of overall survival of patients with *EGFR* Mut + advanced NSCLC who ever received either a TKI (**a**) or gefitinib (**b**) during their entire course of treatment compared with those who did not receive a TKI. KM, Kaplan-Meier; Mut+, mutation-positive; NSCLC, non-small-cell lung cancer; TKI, tyrosine kinase inhibitor

Of those patients who received a TKI/gefitinib before first disease progression, PFS was longer compared with those who did not receive a TKI (Fig. 2a and b). Analysis of OS showed no significant difference between these patient populations (Fig. 2c and d). However, longer OS was reported in those patients who ever received a TKI during their complete therapy course compared with those who never received a TKI: median OS 18.4 vs. 13.6 months; HR 0.53; log-rank *p* = 0.003 (Fig. 3a). A similar outcome was shown for those patients who ever received gefitinib compared with those who never received a TKI: median OS 18.1 vs. 13.6 months; HR 0.55; log-rank *p* = 0.005 (Fig. 3b).

RR was 50.9% overall (Table 2) and was higher in the following groups: female versus male; never smoker versus ever smoker; ever EGFR inhibitor versus never EGFR inhibitor; TKI-sensitive versus TKI-insensitive *EGFR* mutations.

### Pharmacoeconomic endpoints

The three first-line treatment groups comprised chemotherapy (*n* = 90), TKI (*n* = 159), and switch to TKI (*n* = 31). Total cost of treatment was highest for the TKI group (€46,443) and lowest for the chemotherapy group (€27,182). For all three groups, cost of drug was the main expenditure. As a proportion of the total costs, drug costs were higher with TKI and switch therapy (75.5% and 76.7%, respectively) compared with chemotherapy (57.1%). In terms of mean outpatient and inpatient costs, the chemotherapy group had the highest costs and the switch group the lowest (Additional file 1: Table S1).

The number of patients with a documented nursing auxiliary decreased during the course of observation in the chemotherapy group (13.7% vs. 12.7%) and increased in the TKI and switch groups, by 5.4 percentage points (15.5% vs. 20.9%) and 16.1 percentage points (22.6% vs. 38.7%), respectively. The proportion of patients without a nursing auxiliary listed at the final visit was 62.6%, 60.8%, and 51.6% for the TKI, chemotherapy, and switch groups, respectively.

The number of patients with an employment relationship decreased throughout the observation period in all three groups. The biggest changes were seen in the switch group (25.8% to 3.2%), compared with the chemotherapy (28.4% to 8.8%) and TKI (18.2% to 7.5%) groups. However, the chemotherapy group had a higher proportion of patients with an unknown employment relationship at the end of treatment (26.5%) than the TKI (17.1%) and switch (9.7%) groups. At the last visit, the proportions of patients with full-time employment in the chemotherapy, TKI, and switch groups were 4.9%, 5.3%, and 3.2%, respectively.

### Safety

Over half of the patients receiving gefitinib reported at least one AE (58.1%), of which rash, diarrhea, and dry skin were the most common AEs (Table 3) and ADRs. A total of 20 grade 3–5 ADRs were reported, including two patients each with grade 3 rash, diarrhea, and nausea and two grade 4 reactions (diarrhea and thrombosis/thrombus/embolism).

**Table 3:** AEs in patients with *EGFR* Mut + NSCLC treated with gefitinib (≥ 2% of patients)

	*n* (*N* = 222)	%
All	129	58.1
Dermatology/skin		
Rash: acne/acneiform	53	23.9
Dry skin	24	10.8
Nail changes	14	6.3
Pruritus/itching	14	6.3
Dermatology/skin – other	11	5.0
Hair loss/alopecia	9	4.1
Rash/desquamation	8	3.6
Gastrointestinal		
Diarrhea	40	18.0
Nausea	17	7.7
Vomiting	8	3.6
Cardiac general		
Cardiac ischemia/infarction	5	2.3
Constitutional symptoms		
Constitutional symptoms – other	6	2.7
Fatigue (asthenia, lethargy, malaise)	5	2.3
Ocular/visual		
Other	6	2.7
Hemorrhage/bleeding		
Hemorrhage, pulmonary/upper respiratory – nose	5	2.3
Neurology		
Neuropathy: sensory	5	2.3
Pulmonary/upper respiratory		
Dyspnea (shortness of breath)	5	2.3
Renal/genitourinary		
Cystitis^a^	4	1.8

Adverse events by CTC symptoms related to gefitinib and serious adverse events related and not related to gefitinib. AE, adverse event; CTC, Common Toxicity Criteria; NSCLC, non-small-cell lung cancer. ^a^Includes one patient in whom cystitis was not related to gefitinib and was not serious

Serious AEs were reported for 49 patients (22.1%), the most frequent of which were cardiac ischemia/infarction and constitutional symptoms, other (2.3%, each), followed by diarrhea and cystitis (1.8%, each). Eight patients (3.6%) had AEs leading to discontinuation of treatment with gefitinib, including diarrhea (*n* = 4) and nausea (*n* = 2). There were 11 deaths, only one of which was considered to be related to treatment with gefitinib (hemorrhage, pulmonary/upper respiratory – bronchopulmonary not otherwise specified).

## Discussion

To date, the REASON study represents the largest dataset of information on *EGFR* mutations in Caucasian patients with NSCLC. In the REASON study, 10.3% of patients were tested positive for *EGFR* mutations, similar to the European population (12%) in ASSESS, a large multicentre, non-interventional diagnostic study in patients with advanced NSCLC [7].

In patients with *EGFR* Mut + NSCLC who received a TKI (or gefitinib as their TKI) before first disease progression, PFS was prolonged by about three months compared with those who did not receive a TKI. The RR was higher in patients receiving first-line TKI than in those not receiving a TKI (53.2% vs. 45.0%). Median OS was similar between those patients who received a TKI or gefitinib before first disease progression compared with those who did not receive a TKI. These outcomes for PFS, OS, and RR parallel those of clinical trials comparing TKIs with standard doublet chemotherapy regimens [3, 4, 8].

A survival analysis of patients with *EGFR* Mut + NSCLC who ever received a TKI (or gefitinib as their TKI) during the course of their treatment revealed an increase in median OS of approximately five months compared with those who never received a TKI. However, when interpreting these data it should be considered that by virtue of surviving longer, patients may have received a greater number of treatments (including EGFR-TKIs) compared with those patients with poorer prognosis. This may have biased the REASON OS analysis in favor of those patients who ever received a TKI during their entire treatment course (*n* = 242) compared with those who never received a TKI (*n* = 61).

Previous real world studies suggested that patients with *EGFR* Mut + disease who receive targeted therapy survive longer [9, 10]. In contrast the EPICLIN-lung study did not show any benefit, most likely because TKIs were often used without selection for *EGFR* mutation [11]. To date no significant differences in PFS between gefitinib and erlotinib have been reported in real world studies [12, 13].

In the REASON study, first-line treatments for all patients commonly included platinum agents and pemetrexed, similar to the findings from MUTACT (a French observational study on the management of patients with NSCLC adenocarcinoma) [14]. Altogether, 6.2% of patients in the REASON study received gefitinib as first-line treatment, fewer than reported in the MUTACT study (23%). There were also fewer patients with *EGFR* Mut + NSCLC receiving a TKI first-line in the REASON study (56.6%) compared with the MUTACT study (76%). As previously reported, this possibly reflects patients with acute symptoms initiating first-line chemotherapy while waiting for *EGFR* mutation test results and who subsequently switch to an EGFR-TKI once a positive mutation test was confirmed [6]. The proportion of patients with *EGFR* Mut + NSCLC who ever received a TKI during their entire treatment course was 80% (242/303 patients). This is broadly in line with an Asian retrospective cohort study of patients with advanced NSCLC, in which 88% of the patients with *EGFR* Mut + NSCLC received a TKI at some point in their treatment (first-, second-, or third-line) [15]. The majority of patients in REASON with *EGFR* Mut + NSCLC who received an EGFR-TKI first-line were prescribed gefitinib over erlotinib; this could be explained by the regulatory status of the EGFR-TKIs at the time of the REASON study. Gefitinib was approved for use in patients with locally advanced or metastatic NSCLC with activating mutations of EGFR-TK in July 2009, whereas erlotinib was approved as a first-line monotherapy in the same group of patients in September 2011, 2 years after the start of REASON [16, 17]. Pemetrexed was the most commonly used second- and third-line treatment for patients with *EGFR* Mut + NSCLC, followed by erlotinib and gefitinib.

The cost of treating patients during first-line therapy until progression was 40% lower in the chemotherapy group than in the TKI group. For all three groups, drug costs were the main expense, followed by inpatient costs. Drug costs for chemotherapy were around half compared with the TKI and switch groups. However, the highest mean outpatient and inpatient costs were documented for chemotherapy patients. It should be noted that the AE profile of gefitinib in the REASON study was consistent with that described in the Summary of Product Characteristics [18]. At the end of the observation period, more patients in the TKI group did not have a nursing auxiliary listed compared with the chemotherapy group (62.6% vs. 51.6%). Taken together, these data suggest EGFR-TKIs as first-line treatment in patients with *EGFR* Mut + NSCLC results in fewer medical interventions than with chemotherapy. This is supported by a study on the impact of targeted treatment on direct medical costs of patients with advanced NSCLC, which showed targeted agents for patients with *EGFR* Mut + NSCLC lowered the mean monthly medical costs by prolonging survival and diminishing the use of other medical resources [19].

The numbers of patients with employment relationships at the end of the observation period were low in all treatment groups. They were particularly low for switch patients (3.2% vs. 8.8% for chemotherapy and 7.5% for TKI therapy). However, the larger number of patients with an unknown employment relationship at the end of observation in the chemotherapy group compared with the other two groups challenges the interpretation of these data.

## Conclusions

Findings from the REASON study secondary endpoints provide a valuable insight into current treatment patterns, clinical outcomes and resource use in patients with *EGFR* Mut + NSCLC in Germany. In summary, RR, PFS and OS with first-line EGFR-TKI treatment for patients with EGFR Mut + advanced NSCLC are in line with expectations based on previous clinical trials. OS analysis across the entire treatment course reveals a benefit in those patients who ever received an EGFR-TKI vs those who did not, which is in line with other real-world evidence [10]. The cost of first-line EGFR-TKI treatment is more expensive than chemotherapy; however, the highest mean outpatient and inpatient costs were documented for chemotherapy patients, and at the end of the observation period, more patients in the TKI group did not have a nursing auxiliary listed compared with the chemotherapy group.

## Additional file

Additional file 1: Table S1. Treatment costs according to type of first-line treatment received by patients with *EGFR* Mut + NSCLC. (DOCX 15 kb)

## Abbreviations

ADR: Adverse drug reaction
AE: Adverse event
EGFR: Epidermal growth factor receptor
NSCLC: Non-small cell lung cancer
OS: Overall survival
PFS: Progression-free survival
REASON: Registry for the epidemiological and scientific evaluation of *EGFR* mutation status in patients with newly diagnosed locally advanced or metastatic NSCLC
RECIST: Response evaluation criteria in solid tumours
RR: Response rate
TKI: Tyrosine kinase inhibitor
