# Supplementary material for: Treatment decisions, clinical outcomes, and pharmacoeconomics in the treatment of patients with EGFR mutated stage III/IV NSCLC in Germany: an observational study
Source: BMC Cancer. 2018 Feb 5;18:135. doi: 10.1186/s12885-018-4032-3 (PMC5799904; doi:10.1186/s12885-018-4032-3)
Supplement: Additional file 1: Table S1. — Treatment costs according to type of first-line treatment received by patients with EGFR Mut + NSCLC. (DOCX 15 kb) [file 12885_2018_4032_MOESM1_ESM.docx]

**Supplementary Table 1.** Treatment costs according to type of first-line treatment received by patients with *EGFR* Mut+ NSCLC

| **Mean cost ± SD (range), €** | **Chemotherapy^a^ (n = 90)** | **TKI^b^ (n = 159)** | **Switch to TKI^c^ (n = 31)** |
| --- | --- | --- | --- |
| Drug | 15,528 ± 26,801  (83–157,798) | 35,080 ± 28,810  (800–120,430) | 28,049 ± 28,164  (191–93,719) |
| Inpatient | 11,127 ± 12,486  (0–76,772) | 10,895 ± 10,106  (0–52,472) | 8,308 ± 7,455  (0–31,412) |
| Outpatient | 527 ± 866  (0–3,501) | 469 ± 807  (0–4,772) | 210 ± 464  (0–2,175) |
| Total | 27,182 ± 34,086  (252–235,690) | 46,443 ± 34,597  (2,222–158,278) | 36,567 ± 33,683  (860–111,967) |

NSCLC, non-small-cell lung cancer; SD, standard deviation; TKI, tyrosine kinase inhibitor.
^a^All patients receiving cytotoxic chemotherapy or switching to chemotherapy.

**^b^**All patients receiving erlotinib or gefitinib or switching between these plus one patient receiving TKI first-line who switched therapy without documentation of the new therapy.

**^c^**Switch from non-TKI therapy to TKI therapy.
